# Peripheral Blood Transcriptome in Breast Cancer Patients as a Source of Less Invasive Immune Biomarkers for Personalized Medicine, and Implications for Triple Negative Breast Cancer

**DOI:** 10.3390/cancers14030591

**Published:** 2022-01-25

**Authors:** Helena Čelešnik, Uroš Potočnik

**Affiliations:** 1 Faculty of Chemistry and Chemical Engineering, University of Maribor, Smetanova Ulica 17, 2000 Maribor, Slovenia; helena.celesnik@um.si; 2 Center for Human Genetics & Pharmacogenomics, Faculty of Medicine, University of Maribor, Taborska Ulica 8, 2000 Maribor, Slovenia; 3 Department for Science and Research, University Medical Centre Maribor, Ljubljanska Ulica 5, 2000 Maribor, Slovenia

**Keywords:** triple negative breast cancer, transcriptome, peripheral blood, PBMC, gene expression, immune biomarker

## Abstract

**Simple Summary:**

Triple-negative breast cancer (TNBC) is an aggressive and heterogeneous breast cancer (BC) type which is difficult to treat and accompanied by disease recurrence. A better understanding of TNBC and the identification of novel biomarkers is needed to facilitate clinical decisions. Immune-related biomarkers are of particular interest, since immune responses play an important role in BC outcome. Transcriptome studies of peripheral blood cells can help us to understand the systemic immune responses to cancer cells and the mechanisms underlying cancer initiation and progression. They enable the identification of novel immune biomarkers for early cancer detection and personalized BC management and may bring forward new immunotherapy options. Recent transcriptome analyses of peripheral blood cells have delineated novel BC-patient immune subgroups. This categorization has implications for cancer prognosis, the identification of patients likely to benefit from immunotherapy, and treatment efficacy monitoring. Additionally, transcriptome studies have identified TNBC-enriched blood transcriptional signatures that can differentiate TNBC from other classical BC subtypes.

**Abstract:**

Transcriptome studies of peripheral blood cells can advance our understanding of the systemic immune response to the presence of cancer and the mechanisms underlying cancer onset and progression. This enables the identification of novel minimally invasive immune biomarkers for early cancer detection and personalized cancer management and may bring forward new immunotherapy options. Recent blood gene expression analyses in breast cancer (BC) identified distinct patient subtypes that differed in the immune reaction to cancer and were distinct from the clinical BC subtypes, which are categorized based on expression of specific receptors on tumor cells. Introducing new BC subtypes based on peripheral blood gene expression profiles may be appropriate, since it may assist in BC prognosis, the identification of patients likely to benefit from immunotherapy, and treatment efficacy monitoring. Triple-negative breast cancer (TNBC) is an aggressive, heterogeneous, and difficult-to-treat disease, and identification of novel biomarkers for this BC is crucial for clinical decision-making. A few studies have reported TNBC-enriched blood transcriptional signatures, mostly related to strong inflammation and augmentation of altered immune signaling, that can differentiate TNBC from other classical BC subtypes and facilitate diagnosis. Future research is geared toward transitioning from expression signatures in unfractionated blood cells to those in immune cell subpopulations.

## 1. Introduction

Breast cancer (BC) is the most diagnosed cancer in the world and the leading cause of cancer death in women [1]. This disease is very complex and comprises a heterogeneous group of cancer types with distinct pathological features and therapeutic implications. The clinical classification of BC is based on the expression of three clinically validated biomarkers associated with prognosis and treatment options: estrogen receptor (ER), progesterone receptor (PR) and human epidermal growth factor receptor 2 (HER2) [2]. Clinical BC types include *hormone receptor (HR)-positive* (HR+/HER2-, also ER+/PR+/HER2-), *triple positive* (HR+/HER2+, also ER+/PR+/HER2+), *HER2-positive* (ER-/PR-/HER2+, also HER2+), and *triple-negative breast cancer (TNBC)* (HR-/HER2-, also ER-/PR-/HER2-) [2]. Additionally, based on genomic DNA analysis (including copy number, DNA mutations, DNA methylation), gene expression profiling, and protein profiling, phenotypically diverse breast cancers were molecularly characterized and classified into four molecular (intrinsic) groups: *luminal A* (ER+/PR+/HER2-, low proliferation factor Ki67+ (<14%), low grade), *luminal B* (ER+/PR±/HER2±, high proliferation factor Ki67+ (≥14%), high grade), *HER2-enriched* (ER-/PR-/HER2+, any Ki67 level, high proliferation), and *basal-like* (ER-/PR-/HER2-, any Ki67 level, high grade and proliferation, necrosis) [2,3]. Intrinsic BC subtypes partially overlap with clinical subtypes and show significant intragroup molecular heterogeneity [3]. Basal-like tumors are enriched in TNBCs, although 21.4% of TNBCs are not basal-like [4].

Currently, therapy selection and assessment of cancer prognosis are based on the detection of the above-mentioned markers alongside other prognostic and predictive factors (e.g., histology, auxiliary lymph node status, detectable metastatic disease). ER+ tumors represent up to 70% of BC cases and have a good prognosis and survival rate due to their responsiveness to endocrine therapy [2,5]. About 20% of BC cases are characterized by overexpression of HER2, which is correlated with increased aggressiveness. Drugs targeting HER2, such as trastuzumab and lapatinib, have helped to the improve the survival of these patients [6,7]. Conversely, HER2 negativity in ER+ cancers is associated with a better prognosis [2,5]. Triple-negative breast cancer is a complex type of BC with several subtypes [8]. It accounts for 10–20% of all BC cases and is known for its aggressiveness, metastasis, high rates of post-treatment relapse, and poor overall survival [2,5,9,10,11,12]. It is also characterized by high proliferative activity, increased immune cell infiltrate, a basal-like and a mesenchymal phenotype, high genomic instability rate and mutational frequency [13]. Since BC therapy commonly involves drugs that target ER, PR and HER2 expressed by HR-positive and HER2-positive breast cancer cells, the absence of these receptors makes treatment of TNBC challenging and associated with treatment failure and disease recurrence [2,14]. For TNBC, the standard treatment remains conventional chemotherapy, although new treatment approaches such as immunotherapy are emerging [13,15]. Due to the lack of recognized therapeutic molecular targets, there is a pressing need for identifying biomarkers that can improve TNBC treatment and can easily be translated into the clinical setting. Biomarkers involved in immune pathways are of particular interest. For one, the heterogeneous TNBC subtypes that are classified according to tumor gene expression profiles include the “immunomodulatory subtype”, which shows enrichment for genes involved in immune cell processes, such as immune cell signaling, cytokine signaling, antigen processing and presentation, and signaling through core immune signal transduction pathways [16]. For treating this subtype, immunotherapy has been recommended [15]. Secondly, as detailed below, immune responses play an important role in the outcome of TNBC in general [17].

## 2. Immune Mechanisms in Breast Cancer and Importance of Immune Responses in TNBC Prognosis and Treatment

**Figure 1:**
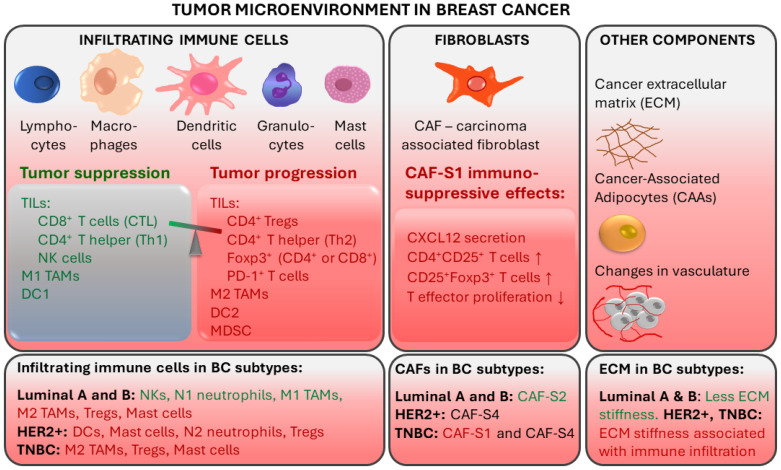
Tumor-associated immune responses in breast cancer. Major players in breast TME include tumor-infiltrating lymphocytes (TILs) and tumor-associated macrophages (TAMs). Non-immune cells such as cancer-associated fibroblasts (CAFs) and cancer-associated adipocytes (CAAs) contribute to an immunosuppressive environment. CTL: cytotoxic CD8^+^ T lymphocytes; Th: CD4^+^ T helper cells; NK: natural killer cells; DC: Dendritic cells; Tregs: regulatory T cells; MDSCs: myeloid-derived suppressor cells. Red text color indicates tumor progression and immunosuppressive effects. Green text color indicates tumor suppression.

While the current clinical decision-making strongly relies on the assessment of receptor expression by tumor cells, it is clear that immune cells and immune signaling are also important in cancer prognosis as well as response to therapy. The tumor microenvironment (TME) is characterized by the presence of innate and adaptive immune cells [18,19]. Studies have identified that tumor-infiltrating lymphocytes (TILs) and tumor-associated macrophages (TAMs) as prominent players in the BC tumor microenvironment. TME also comprises non-cellular components (e.g., inflammation mediating cytokines and growth factors, which correlate with cancer prognosis) and non-immune cells (such as cancer-associated fibroblasts and cancer-associated adipocytes) (Figure 1) [20].

### 2.1. Tumor-Infiltrating Lymphocytes

TILs are immune cells that have left the bloodstream and infiltrated the tumor tissue. They are thought to represent pre-existing antitumor immunity and are clinically meaningful [18]. Elevated tumor TIL presence has been shown to predict a response to neoadjuvant chemotherapy (NAT) in all BC molecular subtypes [21]. TIL levels in the intratumoral stroma have been shown to strongly correlate with good prognosis in TNBC [20,22]. TIL presence at diagnosis of TNBC was positively associated with pathologic response (pCR) to neoadjuvant therapy and disease-free and overall survival after adjuvant chemotherapy, suggesting that anti-tumor immune responses are important in chemotherapeutic sensitivity of BC [23,24]. For every 10% increase in stromal TILs, a risk reduction regarding TNBC recurrence or death was observed [23]. On the other hand, increased TILs was an unfavorable prognostic factor for survival in luminal-HER2-negative patients, suggesting a different biology of the immunological infiltrate in this BC subtype [21]. The prognostic value of TILs in TNBC has also been shown in the absence of adjuvant chemotherapy, identifying a subgroup of early TNBC patients for whom adjuvant systemic therapy might be safely withheld [25,26].

The degree of TIL infiltration assessed by simple hematoxylin and eosin staining of tumor sections is predictive and prognostic in TNBC and HER2-positive BC even without the detailed information on the immune subpopulations of the infiltrate [27]. Nonetheless, deeper immune profiling revealed that the type of TILs, their location and density correlated with BC prognosis and specific outcomes, including pCR [27,28,29]. Better outcomes were associated with infiltrates enriched in CD8^+^ cytotoxic T lymphocytes (CTLs), which are the major effector cell type in BC, and with CD4^+^ T helper cells, natural killer (NK) cells, and dendritic cells (DCs) [29,30,31]. On the other hand, worse prognosis was observed when infiltrates were enriched in regulatory T cells (Tregs), which can suppress immune responses and help to create an immune environment that promotes tumor cell survival and carcinogenesis. Tumor infiltration with Foxp3^+^ Tregs and PD-1^+^ T cells was linked with immune escape [29,31,32]. Furthermore, immature DCs and eosinophils in immune infiltrates were associated with a worse overall survival in TNBC [33]. In TNBC patients who received NAT, pCR was correlated with a higher ratio of CD3^+^:CD68^+^ cells and closer spatial proximity of T cells to tumor cells [28].

In breast TME, immune infiltration is correlated with the presence of hormone receptors and differs between ER+, HER2+, and TNBC tumors. ER-positive breast tumors show an enrichment in NKs and neutrophils, while immune infiltrates in ER-negative breast tumors are enriched in Tregs, activated mast cells (associated with poor prognosis) and M2-macrophages [19].

**Figure 2:**
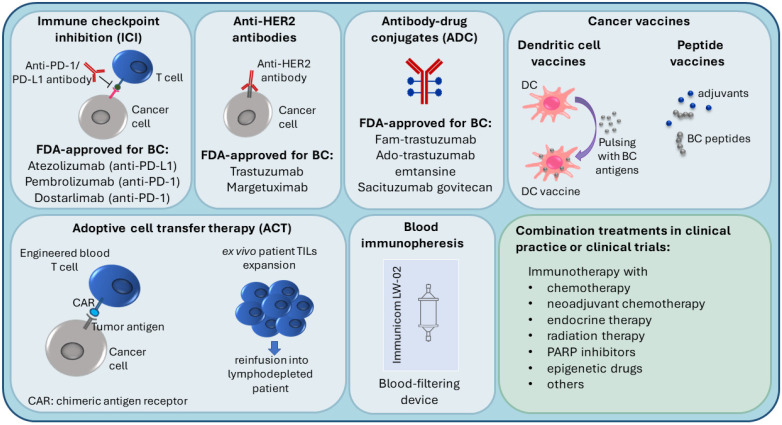
Current immunotherapy strategies in breast cancer treatment.

Tumors can evade recognition by the host immune mechanisms at the level of immune checkpoints. This has led to the identification of immune checkpoint inhibition (ICI) as a promising approach to enhancing antitumor immunity. Immune checkpoint receptor programmed death 1 (PD-1), which is found on T-cells in the TIL infiltrate, functions as a negative regulator of the immune system [34]. Programmed cell death ligand 1 (PD-L1) binds to PD-1, sends a suppressive signal to T cells, and mediates local immune evasion in various types of cancer. Expression of PD-L1 is enriched in basal-like breast tumors compared to other BC subtypes [34,35]. Clinical trials studying PD-1/PD-L1 immune checkpoint inhibition therapy in TNBC have shown positive results and have led to new therapy options for TNBC (Figure 2) [36,37,38,39,40].

### 2.2. Tumor-Associated Macrophages

TAMs derived from peripheral blood monocytes are recruited into the cancer microenvironment and undergo M1/M2 polarization in response to multiple stimuli [41,42]. They are involved in the interaction between the immune system and cancer cells. M1 (CD11c^+^) macrophages have a proinflammatory function, while M2 (CD163^+^) TAMs show immunosuppressive action [43]. The tumor stroma of TNBC and basal-like BC is enriched with CD163^+^ M2-macrophages, which are associated with higher tumor grade and proliferation [44]. In TNBC, TAMs promote tumor growth and progression in various ways: by secreting inhibitory cytokines, by reducing the effector functions of TILs, by promoting Tregs, and by modulating PD-1/PD-L1 expression in the tumor environment [41]. M2 macrophages are prognostic for lower, while M1 macrophages are prognostic for higher overall and disease-free survival [43]. Luminal A tumors contain fewer CD11c^+^ and CD163^+^ cells compared to TNBC [43].

### 2.3. Other TME Components

Fibroblasts are stromal cells involved in supporting tissues by secreting proteins and remodeling the extracellular matrix (ECM). Breast TME contains carcinoma-associated fibroblasts (CAFs), which contribute to tumor progression by secreting tumor-promoting factors (e.g., chemokines and matrix metalloproteinases) [45,46,47]. Specific CAF subsets were detected in different BC subtypes. CAF-S2 is characteristic for luminal-like tumors [48]. The significant presence of CAF-S2 is also seen in normal breast tissue, suggesting that CAFs in luminal BC may be derived from normal resident fibroblasts [47,48]. In aggressive BC subtypes, CAF-S1 and CAF-S4 are characteristic: HER2+ tumors are enriched in CAF-S4, while TNBC tumors have a high presence of CAF-S4 and CAF-S1 [48]. CAF-S1 fibroblasts were found to promote an immunosuppressive environment by increasing the capacity of Tregs to inhibit T effector proliferation, by attracting (via CXCL12 secretion) and retaining CD4^+^CD25^+^ TILs, and by promoting their differentiation into CD25^+^FOXP3^+^ TILs [48].

Breast cancer is associated with extensive remodeling of ECM. Particularly in TNBC and HER2+, stiffening related to collagen deposition and linearization, which is associated with enhanced immune cell infiltration, is observed [49]. In contrast, luminal-like breast cancers undergo less ECM remodeling. Additionally, breast TME contains cancer-associated adipocytes (CAAs), which express proteases that degrade the ECM. CAAs are characterized by reduced lipid content, expression of specific adipokines and proteases, and increased pro-inflammatory cytokine production [47]. Fragmentation of ECM by metalloproteinases and remodeling enzymes can promote the penetration of CAFs [50].

## 3. Immunotherapy in Breast Cancer

Due to higher TIL and TAM levels, increased PD-L1 expression, greater mutational burden, and genomic instability, TNBC and HER2+ breast cancers are considered more immunogenic compared to luminal BC, with the immune responses of these patients affecting the disease outcome [29,37,48,51,52]. Because of this, TNBC and HER2+ patients are most suitable for immunotherapy, which is an important emerging treatment approach in BC (Figure 2), and most of the BC immunotherapy efforts have focused on these subtypes [29,52]. Several targets in immune pathways have been explored [51].

### 3.1. Immune Checkpoint Inhibition (ICI), Anti-HER2 Antibodies and Antibody-Drug Conjugates (ADC)

Active BC immunotherapies have been designed to target the immune checkpoints (such as PD-1/PD-L1) on cancer cells and in the TME. Checkpoint inhibitors used in clinical practice for BC target either the PD-1 receptor or the PD-L1 ligand. The disruption of PD-1/PD-L1 interaction enhances the ability of T cells to attack the tumor [53]. The U.S. Food and Drug Administration (FDA) (https://www.fda.gov/, accessed on 14 November 2021) recently (in 2019) approved atezolizumab (anti-PD-L1 antibody) with paclitaxel as a combination therapy for PD-L1-positive unresectable locally advanced or metastatic TNBC [54]. In 2021, the FDA-approved pembrolizumab (anti-PD-1 antibody) in combination with chemotherapy for high-risk early-stage TNBC and for locally recurrent unresectable or metastatic PD-L1-positive TNBC tumors [55]. Moreover, in TNBC clinical trials, PD-1/PD-L1 inhibitors are being investigated in combination with a number of other treatments, such as radiation therapy (NCT04683679), PARP inhibitors (i.e., DNA damage response inhibitors) [56], anti-CTLA4 (cytotoxic T lymphocyte–associated antigen 4) ICI immunotherapy (NCT03982173), and others [51].

Several immune therapies and immunotherapy combination treatments have received FDA approval for HER2-positive BC. They include trastuzumab (anti-HER2 antibody), margetuximab (anti-HER2 antibody), fam-trastuzumab (antibody-drug conjugate (ADC); combination of trastuzumab and the chemotherapeutic DXd), ado-trastuzumab emtansine (ADC; combination of trastuzumab and the chemotherapeutic emtansine) [57,58,59,60]. Trastuzumab can be used alone or combined with chemotherapy and/or pertuzumab (a HER2 dimerization inhibitor) [58]. ADCs are also being investigated in TNBC [61]. In 2021, the FDA approved sacituzumab govitecan (combination of anti-Trop2 antibody and the chemoterapeutic SN-38) to treat unresectable locally advanced or metastatic TNBC [62].

Luminal BC tumors, which are less immunogenic, are the least likely to respond to immunotherapy. Consequently, fewer immunotherapy studies have been carried out in this subtype [51]. In ER-positive tumors, PD-1/PD-L1 inhibitors are being investigated in combination therapies such as endocrine therapy (NCT02997995) and neoadjuvant chemotherapy (NCT03356860).

### 3.2. Personalized Immunotherapy Approaches

Several novel immunotherapy approaches are currently being tested, including vaccines, cell-based immunotherapies, and immunopheresis.

In peptide-based cancer vaccines, BC-specific antigens are used to stimulate the host immune system. Endogenous antigens (prepared from the patient’s tumor) or exogenous antigens (known to be tumor-specific, for example, HER2 peptide) can be introduced with adjuvants into the patient to stimulate an effector and memory T cell response [63,64,65].

Dendritic cell (DC)-based cancer immunotherapy (i.e., DC vaccination) involves ex vivo pulsing of patient DCs with tumor cell lysates or tumor peptides prior to reinfusion into the patient. Patient DCs are prepared from peripheral blood precursors that are in vitro differentiated into DCs [66,67].

Adoptive cell transfer (ACT) therapies come in different variations. For example, lymphocytes can be isolated from the patient, expanded ex vivo and then reinfused into the patient, who undergoes lymphodepletion to reduce cells that contribute to immunosuppression [64,68]. Another adoptive cell transfer strategy is the use of genetically modified cells; autologous immune cells are removed from peripheral blood, genetically engineered to recognize a specific tumor antigen, and then reinfused into the patient. For instance, in chimeric antigen receptor (CAR) T-cell therapies, T cells are engineered to express a protein that can recognize tumor antigens on the tumor cell and a signaling domain that can turn the T cell on [64,69].

Recent advances in immunopheresis have made it possible to selectively remove specific immune-suppressive molecules from the blood of TNBC patients in order to re-energize the immune system to aggressively fight cancer (NCT04142931) (https://immunicom.com/ (accessed on 14 November 2021)) [70].

Overall, the emergence of immunotherapy has transformed the treatment standard of BC. After the completion of numerous clinical trials, immunotherapy is expected to become even more widespread. Transcriptome studies may prove useful in identifying new therapeutic targets for immunotherapy and novel BC markers that could be useful in disease/treatment monitoring. Additionally, transcriptome analyses may enable further characterization of patient immune responses, which would optimize patient stratification and selection of patients who are likely to benefit from immunotherapy.

## 4. Transitioning toward Less Invasive Immune-Related Biomarkers for Cancer Detection and Prognosis

While tissue-specific biomarkers, including immune-cell infiltration of the tumor, atypical cells, changes in tumor gene expression, and other malignant changes can serve as reliable cancer biomarkers, they have certain limitations. For instance, the invasiveness of biopsy acquisition makes tissue-specific biomarkers ill-fitted for the real-time monitoring of treatment response. Additionally, while TILs may be powerful prognostic biomarkers and have significant predictive value in identifying patients with the highest likelihood of responding to therapy [71], they are not useful for the early detection of cancer or for cancer screening in people with no symptoms. The currently used diagnostic tools such as biopsies and mammography are uncomfortable for patients, can be costly, and may be appropriate only for tumors that have developed to a specific extent [72]. For instance, breast cancer may exist for a while before it is detected by mammography, since the sensitivity of BC screen-testing depends on tumor size, increasing from 26% at 5 mm to 91% at 10 mm tumor size [73]. Furthermore, mammographic sensitivity for BC declines significantly for women with dense breast tissue [74]. Since patient survival rates increase substantially if cancer is identified at the early stages, high sensitivity and specificity of early cancer detection remain among the most important and challenging issues [75]. For early cancer detection, acquiring tissue by invasive means can be difficult to justify, as it comes with certain risks and may not be a good choice for weak patients. Less invasive and more easily accessible methods of biological sample acquisition, such as blood collection, can make early detection more feasible and may increase acceptance among patients, thereby leading to potentially faster diagnosis.

## 5. Circulating Blood Cell Transcriptome as a Source of Less Invasive Breast Cancer Biomarkers

Tumor development and survival involve active crosstalk between cancer cells, normal stromal cells, adjacent tissues, and host immune defense system [72,76,77]. Primary tumors release a range of signaling molecules into their surroundings. Circulating blood cells monitor the physiological state of the body and respond to tumor signals with phenotypic and functional changes, rendering them able to either kill cancer cells or to promote cancer proliferation and metastasis [78,79,80]. While peripheral blood diagnostic and prognostic biomarkers include changes in peripheral blood counts, alterations in gene expression, DNA methylation, miRNA profiles, and other changes [81,82], we will focus on transcriptomic changes in peripheral blood cells (PBCs) of BC patients.

Tumor signals trigger distinctive changes in the transcriptome of circulating blood cells [11,83,84,85]. The resulting gene expression signatures can be clinically useful for the detection and characterization of primary tissue tumors, for cancer prognosis, and for monitoring or predicting the efficacy of therapies [5,72,76,85,86,87]. In peripheral blood cells of BC patients, Dumeaux et al. identified deregulated processes that reflect a deficit in immune functions. They proposed a signature of 50 genes associated with systemic immunosuppression which indicate the presence of BC and classify women with changes other than breast carcinoma (i.e., population-based controls, gastrointestinal and brain cancer patients) as negative [88]. Underexpression of several immune pathways, such as antigen processing and presentation (e.g., downregulation of MHC I molecules), decreased CD4 (which is involved in helper T cell (Th) activation), and impairment of natural killer (NK) cell-mediated immunity was observed [88]. Likewise, in a gene expression study of peripheral blood mononuclear cells (PBMCs), a subset of circulating blood cells comprising immune cells (i.e., monocytes, lymphocytes, NK and dendritic cells), the observation that NK-cell activity is decreased in BC patients compared to controls was confirmed [76]. This was evidenced by lower expression of activating receptors NKp30, NKp46 and 2B4 in BC, although the percentage of NK cells and the proportion of the primary NK cell subsets were similar in both groups [76]. Furthermore, Suzuki et al. described the upregulation of cell differentiation pathways of specific subsets of helper T cells (Th17, Th22, Th9) in PBMCs of BC patients compared to healthy subjects, as well as upregulation of TLR3- and TLR4-induced TICAM1-specific signaling pathway [89]. In colorectal cancer, the subsets Th17, Th22 and Th9 and their cytokines have been reported to contribute to its development [90].

Cytokines are key signaling molecules of intercellular communication in the immune system, known to mediate either stimulatory or inhibitory tumor responses. Dysregulation of circulating cytokines has been identified as an important dissimilarity between BC and healthy subjects [87]. Tumor-elicited cytokines are generated in the TME, from which they spread into the circulation. They can be detected in blood during cancer and have a high potential as biomarkers, particularly for monitoring the severity of cancer and efficacy of drug intervention [91]. Apart from cytokines produced in the TME, peripheral immune cells of cancer patients (even those with localized tumors) can also show dysregulated immune cytokine signaling signatures [91]. For instance, compared to healthy controls, peripheral nucleated blood cells of BC patients have been reported to overexpress a number of proinflammatory factors, such as CXCL1, CXCL2, CXCR4, CCL3, CCL4, IL-8, and others [5]. Cytokines and other biomediators that are upregulated in PBCs of BC patients have been suggested to stimulate the innate peripheral blood immune cells (e.g., phagocytes, granulocytes) to infiltrate TME [5].

Peripheral blood cells of BC patients also show changes in several universal cell programs (i.e., cell metabolism, growth, and proliferation) compared to healthy subjects [88,92]. Deregulation of genes involved in ribosome production and translation control, as well as of various metabolic processes, such as lipid and steroid metabolism, was reported in the peripheral blood of BC patients [84,93].

## 6. PBMC Gene Expression Biomarkers for Classification of Novel BC Subtypes

Considering that PBMCs comprise blood immune cells that mediate the host immune response to tumor cells, peripheral blood profiling can be useful for evaluating the host’s reaction against cancer and offers the possibility for minimally invasive early cancer detection (even before the development of clinical symptoms), thereby distinguishing BC and healthy subjects [94,95,96]. It can also be valuable for predicting tumor progression and for the prognosis of clinical outcome [92,97,98].

In addition to differentiating healthy subjects from those with BC, several studies have attempted to identify the differences in PBMC gene expression *within* breast cancer to distinguish BC subtypes (Table 1) [5,76,89,97]. Important common observations that emerged were that PBMC transcriptomes in BC patients correlate poorly with classical BC subtypes and show substantial heterogeneity [85,89,97]. For instance, a study by Ming et al. involving RNA sequencing of PBMCs from 33 treatment-naïve BC patients (16 luminal A, 6 luminal B, 3 HER2-positive, and 8 TNBC) revealed that the established BC subtypes based on ER, PR, and HER2 were not associated with transcriptome-wide PBMC gene expression profiles [97]. This is not entirely unanticipated, considering that PBMC gene expression represents the *immune reaction* of blood mononuclear cells to the presence of tumor cells. Similar findings—substantial heterogeneity of peripheral blood leucocyte transcriptomes independent of histological type—were reported for lung cancer patients, where despite distinct origins of different histological types, there were no marked differences in influence on the peripheral immune system [99]. In breast cancer, Ming et al. further used unsupervised cluster analysis of PBMC gene expression, which identified two new BC subtypes, each comprising patients from all the established subtypes [97]. The difference between the novel subtypes was their distinctive immune response to tumor, including the activation of immune cells, the regulation and response of the innate and adaptive immune system, and the production of specific antibodies. Important distinct patterns included osteoclast differentiation (which is associated with metastasis) and the interleukin-10 signaling pathway (which is associated with inflammatory processes and tumor immunology). The novel subtypes also had different neutrophil-to-lymphocyte ratios, which indicate the inflammation level [97]. Interestingly, one of the subtypes was enriched in a 28-gene signature (including IFNGR1, IFNGR2, IL1A, IL1B, TLR2, TLR4, FOSL1, and CSF1), which had the ability to predict clinical outcome. Its tissue expression was associated with lower risk of recurrence and better survival of BC, including the basal-like breast cancer subtype, which is enriched in TNBC [97].

Similarly, RNA sequencing of PBMCs from 13 BC patients and 3 healthy volunteers identified BC and healthy subjects as two distinct clusters, whereas the BC group formed two distinct subsets that did not correlate with the classical BC subtypes [89]. These two subsets differed mainly in B-cell receptor immunological pathways and chemoattractant receptor-homologous molecule on Th2 (CRTH2) signaling in Th2 cells. Based on further analysis of immune-activating and immune-inhibitory gene expression patterns in PBMCs, the authors suggested four immunological subgroups; all healthy subjects were in the “lymphocyte retention group” (upregulated CD8A, CD4, CD248, IDO1, IDO2), while BC patients were in “monocyte activating” (upregulated CD14, CD40, CD80, Siglec14, NRP1, TIM3), “T-cell inhibitory” (upregulated PD-L1, PD1, CTLA4, FOXP3, CCR3), or “unknown” group. With regard to TNBC patients, three out of four ER-HER2- patients were in the monocyte-activating group.

Overall, the above findings indicate that PBMC transcriptome in BC patients is influenced by the presence of cancer, not by BC subtypes [89,97]. This is in line with a study by Foulds et al., which combined analyses of PBMC transcriptome and blood immune cell profiling in TNBC and luminal A patients [76]. The overall PBMC immune expression profiles of the two BC subtypes were similar. However, 21% of TNBC patients had a clearly distinct immune expression pattern, enriched in transcripts involved in inflammation. From the coupled immune-cell and transcriptomic profiling, the authors concluded that the peripheral blood immunome in BC is influenced by the presence and stage of cancer, not by molecular subtypes (i.e., is disease-related rather than molecular subtype-specific) [76].

Finally, based on their observations, Ming et al. suggested that PBMC transcriptome-based subtyping could serve as a novel and independent classification for BC patients [97]. However, so far, PBMC transcriptome studies have not converged on a select number of common pathways characteristic for specific PBMC immune breast cancer subtypes, highlighting the need for further studies on larger cohorts.

## 7. Gene Expression Biomarkers in Nucleated Blood Cells of TNBC Patients

**Figure 3:**
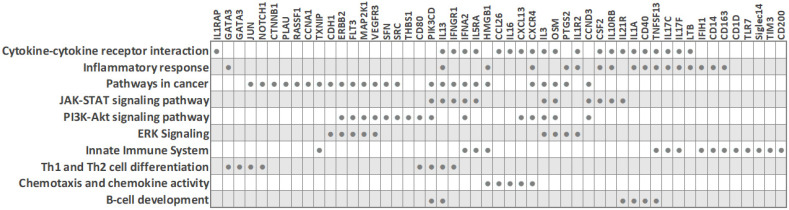
Biological processes and transcripts significantly deregulated in peripheral blood cells of TNBC patients compared to other BC subtypes (based on PBC transcriptome studies). The filled circles mark the transcripts that are differentially expressed in TNBC.

Due to the need for quality biomarkers for TNBC because of its aggressive nature and limited therapeutic options, it is not surprising that several studies have focused on identifying expression differences in peripheral blood cells between TNBC patients and other classical BC subtypes. Despite the described heterogeneity of PBMC transcriptomes in breast cancer, some characteristic molecular features for the TNBC subtype have been reported (Figure 3 and Table 2), as detailed below. The main common observation was that an extensive immune response and tumor-related inflammation are strongly involved in TNBC.

### 7.1. PBMC Transcriptome of TNBC (ER-/PR-/HER2-) versus Hormone-Dependent BC (ER+/PR+/HER2-)

Foulds et al. profiled 770 immune-related genes in 14 TNBC and 9 luminal-A breast cancer patients [76]. Reminiscent of other studies, the immune transcriptome revealed a subgroup of three TNBC patients that had a unique immune expression profile, showing differential expression of genes functionally related to inflammation, including CD163 (a scavenger receptor involved in the resolution of inflammation), cytokine receptors such as IFNGR1 (CD119), IL21R, IL1R2, FLT3, IL1RAP, TXNIP (which promotes anti-inflammatory macrophages), and HMGB1 (which is upregulated under inflammatory conditions). Moreover, the combined low tissue expression of CD163 and CXCR4 and high tissue expression of THBS1 significantly correlated with a high risk of recurrence and poor survival rate in TNBC [76]. The authors suggested that the emergence of a distinct PBMC immune transcriptome subgroup may have implications for clinical decisions.

Balacescu et al. studied the transcriptome of nucleated blood cells from 29 treatment-naïve BC patients with invasive ductal carcinoma and seven healthy controls. The microarray analysis delineated two distinct clusters corresponding to BC and controls [5]. However, within the BC cluster, which included 14 TNBC (ER-/PR-/HER2-) and 15 hormone-dependent (ER+/PR+/HER2-) patients, the expression patterns were mixed and did not cluster according to the ER/PR status, in line with reports by other researchers. Even so, the ER-/PR-/HER2- and ER+/PR+/HER2- groups did show some individual differences in molecular profiles, and a specific 34-gene signature for TNBC, which was able to distinguish TNBC from both hormone-dependent patients and healthy controls, was identified [5]. The nucleated blood cell transcriptome indicated a strong involvement of tumor-related inflammation and immune response in TNBC. Pathway analysis revealed that TNBC was associated with altered systemic immune-related pathways, including chemokine signaling, IL-8 signaling, and communication between innate and adaptive immune cells. Importantly, this dysregulation correlated with increased inflammation and necrosis in the primary tumors. IPA upstream regulator analysis predicted AREG as the upstream regulator in TNBC (with downstream targets AREG/AREGB, CXCR4, EGR1, FOS, PLAU, and PTGS2), while two upstream regulators (AREG and F7) were predicted for hormone-dependent BC. Of note, since the modulation of systemic immune-related pathways was detected in TNBC, the authors concluded that immunotherapy may be a synergistic approach to chemotherapy for this cancer type, which is in line with the findings from other researchers. For example, Suzuki et al. assessed that gene expression profiling in PBMCs could help to obtain an immunological insight with clinical utility for BC—it could be useful for observing the immune reaction related to cancer progression and for monitoring the efficacy of immune-related cancer therapy [89].

Immunological profiling of 84 inflammatory molecules and their receptors in PBMCs of treatment-naïve ductal breast carcinoma patients, including 23 TNBC (ER-/PR-/HER2-) and 17 Her2-luminal (ER+/PR+/HER2-), revealed that TNBC patients had altered expression of PBMC genes associated with immunological status and presented with lower counts of lymphocytes and eosinophils than the ER+/PR+/HER2- patients [12]. Downregulated PBMC genes in TNBC included interleukins (IL13, IL16, IL17C, IL17F, IL1A, IL3), the interleukin receptor IL5RA, cytokines (CSF2, OSM, TNSF13) and the chemokine CCL26. On the other hand, interleukin receptor IL10RB, the chemokine CXCL13 and the cytokine IFNA2 were upregulated in TNBC. IFNA2 and IFN-alpha upregulation suggested the activation of downstream signaling cascade through IL10RB receptors. Overall, the hormone-receptor (ER/PR) expression on HER2-negative breast tumors was associated with distinct systemic PBMC-associated cytokine profiles. These may help us to better understand individual tumor subtypes and open up new possibilities for future immunotherapeutic interventions [12].

In addition to the link between tumor hormone-receptor expression and the systemic cytokine response, the immune activity at the tumor site is also associated with the systemic response (SR). While a high inflammatory SR is observed in both ER-/HER2- and ER+/luminal B patients, systemic inflammation is associated with immune activity at the tumor site depending on subtype. Specifically, ER-/HER2- patients with low immune activity at the tumor site show a high inflammatory SR, while ER+/luminal B patients with high immune activity at the tumor site have a high inflammatory SR [85].

### 7.2. PBMC Transcriptome of TNBC (ER-/PR-/HER2-) versus Her2-Overexpressing BC (ER-/PR-/HER2+)

Tudoran et al. studied whether expression of HER2 on hormone-receptor-negative tumor cells influences the transcriptome of nucleated PBCs. An investigation of 84 breast-cancer associated genes in 18 TNBC (ER-/PR-/HER2-) and 12 Her2-overexpressing (ER-/PR-/HER2+) BC patients revealed 15 genes that were differentially expressed, even though HER2- and HER2+ groups had comparable counts of nucleated cells in blood [11]. Fourteen genes were downregulated in TNBC, including cell cycle regulators (CCNA1, JUN, MKI67, RASSF1, SFN), cell adhesion molecules (CDH1, CTNNB1, HER2), transcription factors (CTNNB1, GATA3, HIC1, JUN, NOTCH1) and signal transducer GLI1. On the other hand, the cell cycle regulator cyclin A1 was upregulated in TNBC [11]. Network analysis indicated that these genes are interconnected, regulate each other, and participate in cancer progression and the modulation of immune signaling. Since fine-tuned engagement of immune responses is needed in favorable treatment response, the altered immune signaling in peripheral blood cells of TNBC patients may contribute to their low treatment response rates, and the authors suggested that baseline monitoring of the immune status may help in treatment response prediction. Interestingly, although HER2-negative expression is characteristic of TNBC tumor cells, decreased HER2 expression was also observed on white blood cells from TNBC patients, in line with previous reports that suggested a correlation between tumor and blood HER2 expression levels [11,100,101].

## 8. Peripheral Blood Cell Transcriptome and Therapy Response and Prognosis in TNBC Patients

The identification of patient immune pathways that are specifically altered in individual BC subtypes can potentially lead to facilitating the selection of the optimal treatment. This holds implications for TNBC, known for treatment nonresponse and poor prognosis.

Recent work has identified NRP-1 (a non-tyrosine kinase receptor) and its interacting molecules as possible drug targets and as biomarkers for predicting poor prognosis in BC [102,103,104]. NRP-1 is involved in primary immune response initiation by mediating the interaction between dendritic cells and resting T cells [105]. Moreover, accumulating evidence indicates that NRP-1 has a role in promoting cancer due to its involvement in the evasion of immune surveillance [106]. Tumor tissue NRP-1 and its soluble isoforms in plasma are upregulated in advanced nodal and metastatic BC, and tumor tissue NRP-1 expression is increased in TNBC compared to other subtypes. In contrast, in PBMCs of BC patients, a downregulation of NRP-1 and its interacting molecules SEMA4A and SNAI1 is seen [102]. This decrease in BC compared to healthy subjects suggests that the interaction of these molecules on PBMCs may not participate in tumor immune evasion but rather may play a protective role against BC. This is supported by evidence that SEMA4A and SNAI1 expression on PBMCs declines with increased tumor size and overall disease stage. Importantly, PBMC upregulation of VEGFR3 and PLXNA (i.e., co-receptors of NRP-1) can distinguish TNBC from other BC subtypes, suggesting their expression on PBMCs may have potential to determine BC cases susceptible to immunotherapy [102]. Altogether, the differences in plasma, tissue and PBMC profiles suggest that NRP-1 has multiple cell type-specific roles in BC, being a risk factor in plasma and tumor tissue and a protective factor in PBMCs [102]. However, Suzuki et al. reported that NRP-1 was elevated in PBMCs of ER-HER2- patients in the “monocyte-activating” immunological subgroup, and therefore further elucidation of NRP-1 in BC is warranted [89].

Neoadjuvant chemotherapy (NAC) is the standard care for a subset of BC patients. Recent advances in immunotherapy in combination with chemotherapy for TNBC have brought forward the need to understand how chemotherapy influences local and systemic immune responses. Axelrod et al. reported that during chemotherapy, increased expression of several immune-related genes and groups of genes (particularly related to T-cells) in the tumor tissue is associated with improved prognosis in TNBC, but not other BC subtypes [107]. Moreover, in peripheral blood of TNBC patients after NAC therapy, high expression of cytolytic markers was detected by single-cell RNA sequencing of the CD8+ PD-1HI population (enriched in tumor-reactive T-cells), and a substantial increase in cytokines expressed by this population was measured [107]. The gene expression signature of immune activation and cytotoxicity (FGFBP2 + GNLY + GZMB + GZMH + NKG7 + LAG3 + PDCD1 − HLA-G) in CD8+ PD-1HI T-cells of TNBC patients and in post-NAC whole blood of BC patients was associated with persistent disease following chemotherapy and disease recurrence after surgery [107,108]. These findings highlight that local and systemic immune-related signatures may help to identify patients with good prognosis following NAC and those likely to benefit from additional immunotherapeutic approaches.

Gene expression is strongly influenced by DNA methylation, an epigenetic modification associated with transcriptional silencing. Tumor suppressor genes (e.g., BRCA1, ATM) are often hypermethylated in patient tumors and in peripheral blood [109,110]. Targeted and genome-wide DNA methylation studies have identified specific methylation changes associated with increased breast cancer risk and BC prognosis [111]. For instance, BRCA1 promoter methylation in peripheral blood was found to correlate with an increased risk of developing TNBC (but not ER+ BC) and was also associated with high-grade tumors [110]. Interestingly, BRCA1 promoter methylation in PBCs correlated with an increased risk of developing BC even in non-carriers of BRCA1 mutation [112,113]. Methylation is also important during cancer treatment, where it is linked with drug resistance. Treatment with chemotherapeutics or immunotherapy was shown to cause acquired methylation-associated resistance in cell models, and therapy resistance was also observed in BC patients [114,115]. The use of epigenetic drugs (e.g., DNMT inhibitors) is being tested in combination with chemotherapy, immunotherapy, and other treatments to overcome epigenetic alterations and treatment resistance (Figure 2) [109,116].

## 9. Future Outlook and Perspective

The aim of this review was to explore the possible diagnostic and clinical utility of gene expression changes that occur in peripheral blood cells of BC patients, particularly the TNBC subgroup.

**Figure 4:**
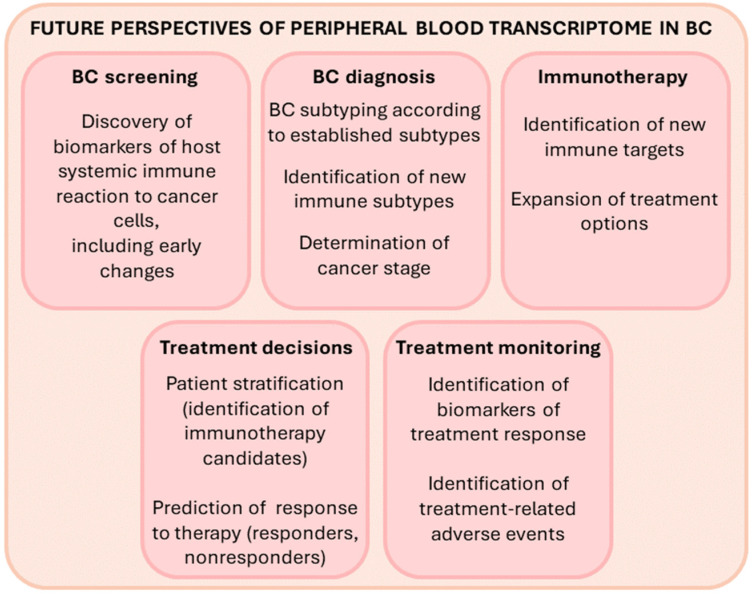
The potential uses of PBC transcriptome analyses for precision medicine in the future.

Since PBC expression changes reflect the immune reaction of peripheral blood cells to the presence of cancer cells, transcriptome analyses of PBCs could be a useful diagnostic tool for early cancer detection and screening (Figure 4). Identifying new noninvasive PBC biomarkers is of great value because early cancer diagnosis is critical for the success of cancer treatment and for patient survival. Transcriptome analyses of PBCs could also be a useful diagnostic tool for personalized management of BC. Multiple recent whole transcriptome studies in peripheral blood cells of BC patients revealed that PBMC expression signatures clearly delineate distinct and novel patient subsets (Table 1) [76,89,97]. A few reports also described specific PBC gene expression changes that can differentiate TNBC from other classical BC subtypes (Table 2 and Figure 3) [5,11,12,76,89,102]. Patient stratification into BC subtypes with distinct peripheral immune responses to the tumor may have important implications for optimal patient treatment and clinical outcome [89,97]. It could enable the selection of patients that would benefit from immunotherapy. For example, Suzuki et al. classified BC patients into four PBMC immune subtypes. One of them, the “T-cell inhibitory gene signature”, showed upregulation of LAG3, CTLA4, FOXP3 and PD-1, which are known as therapeutic targets for current ICI therapy [89]. Accordingly, the authors suggested that ICI therapy may be effective in this patient immune subtype. Moreover, analyzing the expression of immune-related genes in the peripheral blood may predict the response to chemotherapy and serve as a biomarker for those BC patients who would benefit from the combination of chemotherapy and immunotherapy (as opposed to chemotherapy alone) [107,108]. Importantly, patient selection by analyzing PBC gene expression presents a less invasive and more cost-effective method than the methods currently in use. For instance, the selection of BC patients for treatment with atezolizumab currently requires analysis of PD-L1 expression in tumor tissues. It is known that PD-L1 is expressed by tumor cells or immune cells. Masuda et al. reported that in BC patients, PD-L1 expression in blood is correlated with that in tumor tissues and suggested that blood PD-L1 expression could be used as a biomarker to predict the response to atezolizumab [117]. In the future, identification of novel noninvasive immune biomarkers holds promise to expand immunotherapy options and identify predictors of response to therapy, which could facilitate treatment monitoring.

Taken together, transcriptome analyses of PBCs hold promise in advancement of diagnosis and management of BC. Still, several open issues remain. So far, such studies have included a relatively low number of BC patients, and further investigations in larger prospective settings and different ethnic populations will help to determine the consistency of transcriptome results. Additionally, it is still under investigation as to how early during tumor development gene expression changes in blood cells can be detected. Furthermore, while it would clearly be beneficial in the clinical setting to make diagnoses and clinical predictions by analyzing unfractionated peripheral blood cells, this may mask specific immune signatures from immune cell subtypes [76]. PBCs include a spectrum of cell types that may vary in numbers between subjects, therefore changes in blood gene expression may represent altered proportion and/or altered gene expression of distinct cellular populations [76,118]. Thus, gene expression analyses of homogenous cell populations are more likely to be informative and useful [119]. For example, the PBMC fraction consists predominantly of lymphocytes (70–90%), mostly T cells, which encompass a 2:1 ratio of CD4+ T cells (consisting of Treg and Th cells) and CD8+ cytotoxic T cells [120]. It is possible that changes in one cell population (e.g., the CD4+ T cell fraction) may drive changes in the total PBMC expression levels. On the other hand, the altered population distribution of blood cells could cause the differences in transcript levels. For instance, lower counts of lymphocytes and eosinophils were measured in TNBC patients compared to hormone-responsive ER+/PR+/HER2- patients [12].

Another thing to consider when analyzing the transcriptome of peripheral blood cells in cancer patients is that some of the detected gene expression differences may come from circulating tumor cells (CTCs)—the cells shed by the tumor into the bloodstream. However, the contribution of CTCs to total gene expression is likely very small, as deduced from measuring the expression of cytokeratin-19 (used as a marker for detecting CTCs) in PBMCs of BC patients [11]. Moreover, in the early stage of BC, CTCs could not be detected in the peripheral blood of BC patients; they were detected in 2% of stage II and 9% of stage III patients [92].

Studying homogenous blood cell subpopulations could increase comparability between different studies. So far, research groups have used various techniques to obtain peripheral blood nucleated cells (e.g., gradient centrifugation, lysis of erythrocytes in whole blood). However, the findings obtained by different techniques are not entirely comparable. Overall, studies indicate that transcriptome analyses would be stronger if the blood cell population responsible for the differential gene expression was identified and analyzed.

Future technical advances are expected to help further expand our understanding of transcriptional changes that occur in peripheral blood of BC patients. While cost-effective and easy-to-perform standard methods (e.g., qRT-PCR, PCR Arrays) are used in the clinical setting for detection of validated transcript biomarkers in cancer patients, more powerful transcriptome technologies are used for biomarker discovery. Conventional technologies such as PCR arrays and microarrays make it possible to examine hundreds or thousands of transcripts simultaneously. However, they can only analyze predefined transcripts. Moreover, the dynamic range of microarrays is relatively narrow (i.e., transcript detection is limited by the background noise on the lower end and signal saturation at the higher end) [121,122]. RNA sequencing (RNA-seq) is an advanced technology that enables deep gene expression analysis with wider dynamic range and higher sensitivity. It facilitates the discovery of novel transcripts and splicing variants, fused transcripts, rare transcripts, single nucleotide variants and indels [123]. Nonetheless, both microarrays and RNA-seq are bulk transcriptomic analyses. That is, they rely on RNA extraction from a pooled population of cells, which may fail to identify rare cell types or differences between cells, and limits the understanding of individual cell type phenotypes [122]. These limitations can be overcome by the newest technologies such as single-cell RNA-Seq (scRNA-Seq), which enables transcriptome analysis at the single-cell level and identification of phenotypic and functional heterogeneity among single cells [124]. While there is room for improvement even with this technology (e.g., in terms of scalability and cost), scRNA-Seq is expected to facilitate breakthroughs in biomarker discovery and the understanding of immune mechanisms.

## 10. Concluding Remarks

Overall, our understanding of the changes in PBC transcripts and the relationship between breast cancer and immune surveillance has broadened. The continued improvement in our knowledge of immune responses will help us to identify new TNBC predictive and prognostic markers. This will enhance minimally invasive early cancer detection and the development of novel innovative treatments aimed at immune system modulation to render it more combative towards the tumor. In the future, analyses of immune gene expression in the blood will help to inform clinical decisions, including the potential use of immunotherapy.

## Figures and Tables

**Table 1:** Breast cancer immune subsets according to immunological properties of peripheral blood cells.

Study Cohort	Methodology	Novel BC Immune Profiles Based on PBMC Transcriptome	Reference
33 BC (8 TNBC, 16 luminal A, 6 luminal B, 3 HER2-positive)	RNA-seq	Two new BC subtypes differing in the activation of immune cells, regulation and response of innate and adaptive immune system, and antibody production; distinct immune cell proportions (lymphocytes and neutrophils); distinct immune patterns, with altered pathways including myeloid leukocyte activation, osteoclast differentiation and interleukin-10 signaling. Twenty-eight-gene signature enriched in one subtype: TYROBP, IFNGR1, GAB2, TNFRSF1A, PTGS2, NFKB2, NFKBIA, SIRPB1, NFKBIB, RELB, IL1A, IL1R1, IL1B, TLR4, TLR2, FCGR2A, IFNGR2, FCGR3B, JUNB, FOSL1, JUN, SOCS3, SIRPA, CR1, LILRB3, LILRA2, LILRA6, CSF1	[97]
13 BC (4 ER+/HER2-, 2 ER+/HER2+, 3 ER-/HER2+, 4 ER-/HER2-) and 3 healthy subjects	RNA-seq	Two new BC subsets differing in B-cell receptor immunological pathways (Bcl-XL, EGR1, p70 S6 kinase 1, Bcl-10, calcineurin A (catalytic), SOS, calmodulin, SHIP, PI3K reg class IA, IKK-alpha, and TAK1 (MAP3K7)) and CRTH2 signaling in Th2 cells (Bcl-XL, calcineurin A (catalytic), calmodulin, PKC, Apaf-1, and G-protein). Additionally, based on the subset of immune activation- and immune checkpoint-related genes, 4 immunological subgroups suggested: (1) monocyte-activating (CD14, CD40, CD80, Siglec14, NRP1, and TIM3) (included 3 of 4 ER-HER2- patients), (2) lymphocyte retention (CD8A, CD4, CD248, IDO1, and IDO2 (included all healthy controls), (3) T-cell inhibitory (PD-L1, PD1, CTLA4, FOXP3, and CCR3), (4) other	[89]
23 BC (14 TNBC and 9 luminal-A)	Pan-Cancer Immune Profiling Panel, 770 genes	Among all BC patients, a distinct group of 3 patients in the TNBC cohort showed changes in transcripts predominantly involved in inflammation; *upregulated:* IL1R2, THBS1, CD163, FLT3, MFGE8, IFNGR1, IL1RAP, CXCR4, TXNIP, TFRC, CD1D, CCND3, MAP2K1, HMGB1; *downregulated:* CLEC4C, TLR7, LTB, IL21R, IFIH1, PIK3CD	[76]

**Table 2:** Peripheral blood transcriptome studies describing gene expression signatures in TNBC patients.

Study Cohort	Methodology	Gene Signatures in TNBC Patients	Reference
13 BC (4 ER+/HER2-, 2ER+/HER2+, 3 ER-/HER2+, 4 ER-/HER2-) and 3 healthy subjects	RNA-seq	Distinct transcriptome in 3 ER-HER2- patients (i.e., monocyte activating immune subgroup): *upregulated* CD14, CD40 (TNFRSF5), CD80, Siglec14, NRP1, TIM3	[89]
29 BC (14 TNBC and 15 hormone-dependent (ER+/PR+/HER2-)) and 7 healthy subjects	Microarray analysis	Thirty-four-gene TNBC signature (distinguishing TNBC from both ER+/PR+/HER2- and healthy controls); *downregulated genes:* RNU105A, SCARNA5, CD200, SNORA53, BICC1, KLHL31, SNORA81, FAM86DP, SNORD3B-1, RNU2-2, LMAN1, STXBP4, MGC57346, MAP7D2, CCDC39, SNORD15A, SNORD3B-1, ZNF3, SNORD17, SNORA12, NT5E, SNORA74A, NT5E, SCARNA6; *upregulated genes:* PLAU, LOC100128175, ITPK1-AS1, ALPK3, C10orf105, ASAP1-IT1	[5]
23 BC (14 TNBC and 9 luminal-A)	Pan-Cancer Immune Profiling Panel, 770 genes	Distinct transcriptome in 3 out of 14 TNBC patients; upregulated genes: IL1R2, THBS1, CD163, FLT3, MFGE8, IFNGR1, IL1RAP, CXCR4, TXNIP, TFRC, CD1D, CCND3, MAP2K1, HMGB1; downregulated genes: CLEC4C, TLR7, LTB, IL21R, IFIH1, PIK3CD	[76]
40 BC (23 TNBC and 17 luminal (ER+/PR+/HER2-))	Human Inflammatory Cytokines and Receptors PCR Array, 84 genes	*Downregulated in TNBC:* interleukins (IL13, IL16, IL17C (IL21), IL17F, IL1A, IL3), interleukin receptor (IL5RA), cytokines (CSF2, OSM, TNSF13), chemokine (CCL26); *upregulated in TNBC:* interleukin receptor (IL10RB), chemokine (CXCL13), cytokine (IFNA2)	[12]
30 BC (18 TNBC and 12 ER-/PR-/HER2+)	Human Breast Cancer PCR Array, 84 genes	*Downregulated in TNBC:* ERBB2, RASSF1, CDH1, MKI67, GATA3, GLI1, SFN, PTGS2, JUN, NOTCH1, CTNNB1, KRT8, SRC, and HIC1; *upregulated in TNBC:* CCNA1	[11]
70 BC (8 TNBC, 5 luminal A, 20 luminal B, 19 luminal B-like, 13 HER2, 5 unknown) and 50 healthy controls	qRT-PCR	*Upregulated in TNBC compared to non-TNBC subtypes:* VEGFR3 and PLXNA1 (co-receptors of NRP-1)	[102]
